# A Fundamental Regulatory Mechanism Operating through OmpR and DNA Topology Controls Expression of *Salmonella* Pathogenicity Islands SPI-1 and SPI-2

**DOI:** 10.1371/journal.pgen.1002615

**Published:** 2012-03-22

**Authors:** Andrew D. S. Cameron, Charles J. Dorman

**Affiliations:** Department of Microbiology, Moyne Institute of Preventive Medicine, School of Genetics and Microbiology, Trinity College Dublin, Dublin, Ireland; Universidad de Sevilla, Spain

## Abstract

DNA topology has fundamental control over the ability of transcription factors to access their target DNA sites at gene promoters. However, the influence of DNA topology on protein–DNA and protein–protein interactions is poorly understood. For example, relaxation of DNA supercoiling strongly induces the well-studied pathogenicity gene *ssrA* (also called *spiR*) in *Salmonella enterica*, but neither the mechanism nor the proteins involved are known. We have found that relaxation of DNA supercoiling induces expression of the *Salmonella* pathogenicity island (SPI)-2 regulator *ssrA* as well as the SPI-1 regulator *hilC* through a mechanism that requires the two-component regulator OmpR-EnvZ. Additionally, the *ompR* promoter is autoregulated in the same fashion. Conversely, the SPI-1 regulator *hilD* is induced by DNA relaxation but is repressed by OmpR. Relaxation of DNA supercoiling caused an increase in OmpR binding to DNA and a concomitant decrease in binding by the nucleoid-associated protein FIS. The reciprocal occupancy of DNA by OmpR and FIS was not due to antagonism between these transcription factors, but was instead a more intrinsic response to altered DNA topology. Surprisingly, DNA relaxation had no detectable effect on the binding of the global repressor H-NS. These results reveal the underlying molecular mechanism that primes SPI genes for rapid induction at the onset of host invasion. Additionally, our results reveal novel features of the archetypal two-component regulator OmpR. OmpR binding to relaxed DNA appears to generate a locally supercoiled state, which may assist promoter activation by relocating supercoiling stress-induced destabilization of DNA strands. Much has been made of the mechanisms that have evolved to regulate horizontally-acquired genes such as SPIs, but parallels among the *ssrA*, *hilC*, and *ompR* promoters illustrate that a fundamental form of regulation based on DNA topology coordinates the expression of these genes *regardless* of their origins.

## Author Summary

DNA is often considered to be a passive carrier of genetic information, but in fact DNA is an active participant in coordinating the expression of the genes it carries. This is because DNA is a dynamic molecule that can assume a wide range of topologies, and this has a direct impact on the formation of the protein–DNA complexes that drive gene expression. In a bacterium, the chromosome is supercoiled to variable levels according to environmental conditions, and supercoiling in turn governs the topology of gene promoters. Thus DNA supercoiling is able to transduce environmental signals to regulate promoter output. A previous study found that the intestinal pathogen *Salmonella enterica* may use changes in DNA supercoiling to detect when it has entered host immune cells, allowing the bacterium to induce the pathogenicity genes it requires to evade killing by macrophage. In dissecting the underlying molecular mechanisms, we have found that changes in DNA supercoiling also upregulate other key pathogenicity genes, and we have identified the proteins involved in this gene regulatory process. These findings indicate that a fundamental level of gene control arising from the interplay between protein transcription factors and DNA topology regulates *Salmonella* pathogenicity.

## Introduction

*Salmonella enterica* is a facultative intracellular pathogen of the mammalian gut. After passing through the diverse environments of the stomach and digestive tract, *S. enterica* can invade host epithelial cells to gain access to internal tissues where it can persist inside macrophage [1]. The *Salmonella* pathogenicity islands 1 and 2 (SPI-1 and SPI-2) encode type three secretion systems (T3SS) and effector proteins that enable the manipulation and invasion of host tissues [2], [3]. SPI-1 genes are expressed primarily in the intestine during the early stages of invasion, followed by a decrease in SPI-1 expression and an increase in SPI-2 expression inside epithelial cells, and finally SPI-2 expression predominates once *S. enterica* has crossed the epithelium and resides in macrophage vacuoles [4]. Despite this apparently reciprocal pattern of expression over the course of invasion, both gene islands are co-regulated by many of the same global regulatory proteins. For example, SPI-1 and SPI-2 genes are strongly repressed by the nucleoid-associated protein H-NS, a highly-abundant protein that blocks and traps RNA polymerase at gene promoters by forming repressive nucleoprotein complexes [5], [6]. SPI-1 and SPI-2 also share the transcriptional activators FIS and OmpR. FIS is required for full activation of both SPI-1 and SPI-2 genes in laboratory conditions [7], and Δ*fis* mutants are attenuated for virulence in mice [8] and show reduced survival in macrophage [9]. OmpR is a well-characterized direct transcriptional activator of the SPI-2 *ssrAB* promoter [10], and Δ*ompR* mutants are attenuated [11], but the role of OmpR in SPI-1 gene expression has remained ambiguous [2]. It has been recently discovered that together OmpR and FIS drive low-level transcription of SPI-2 in the intestinal lumen, an environment classically thought to be the exclusive domain of SPI-1 [12].

**Figure 1:**
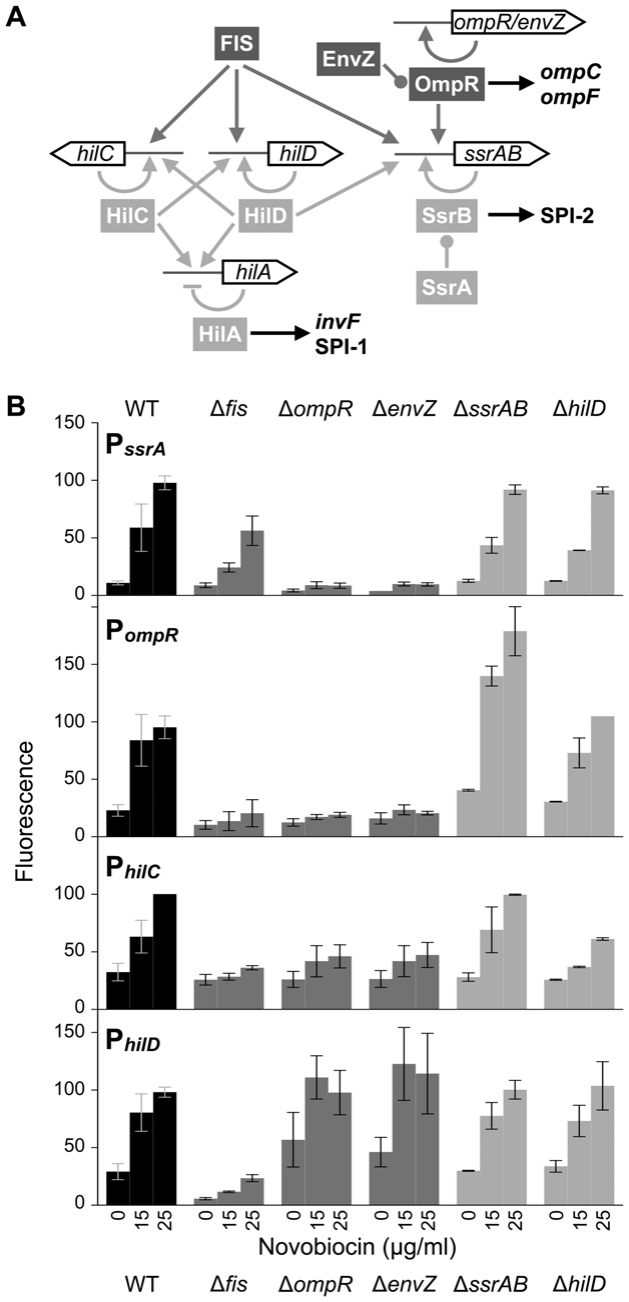
Transcriptional control of SPI-1 and SPI-2 gene expression. A) Schematic of the regulatory connections at the gene promoters described in the text. Global regulators (dark grey) and local SPI-encoded regulators (light grey) are highlighted. Arrows indicate a positive regulatory effect, perpendicular bars indicate a repressive regulatory effect, and rounded bars indicate activation by phosphorylation. B) Expression of the P*_ssrA_:gfp*, P*_ompR_:gfp*, P*_hilC_:gfp*, and P*_hilD_:gfp* transcriptional reporter fusion in response to increasing concentrations of novobiocin (0, 15, and 25 µg/ml) in different genetic backgrounds. Fluorescence values are percentages relative to wild type cells (WT) treated with 25 µg/ml novobiocin; variation in WT values at 25 µg/ml novobiocin indicate differences between replicate experiments conducted on the same day. The mean and standard deviation of 3 to 10 biological replicates are plotted.

By regulating expression of SPI-encoded transcription factors, H-NS, FIS, OmpR, and other global regulators sit atop a hierarchical network that integrates diverse environmental and physiological cues. SPI-encoded transcription factors fine-tune these global inputs to control precisely the dosage of T3SS and effector protein production [13]. SPI-1 encodes four AraC-like transcription factors: HilA, HilC, HilD, InvF. Through a complex feedback and feedforward mechanism, HilC and HilD control their own and each other's transcription, and together activate transcription of *hilA* [14], [15], [16] (Figure 1A). HilA in turn activates *invF* and the genes encoding the T3SS and effector proteins [17]. Additionally, there is crosstalk between SPI-1 and SPI-2 through which HilD induces expression of *ssrAB* [18] (Figure 1A). Unlike SPI-1, SPI-2 encodes a single cognate regulator. Here, an unidentified signal causes the sensor kinase SsrA to phosphorylate the DNA binding protein SsrB, which in turn activates transcription of SPI-2 T3SS and effector genes [3].

SPI-1 and SPI-2 are among the best-studied genetic systems in bacteriology, yet their complex regulation has meant that the mechanisms that integrate the myriad of regulatory signals have remained enigmatic. Even less clear are the contributions made by DNA topology to the interactions and architecture of the nucleoprotein complexes that form at SPI promoters. Several lines of evidence implicate altered DNA supercoiling in coordinating SPI gene expression during invasion. The *invA* gene in SPI-1, which encodes an effector protein, is repressed by relaxed DNA supercoiling [19]. Conversely, *ssrA* expression is induced by relaxation of DNA supercoiling [9]. *S. enterica* DNA is highly supercoiled in low oxygen environments but is more relaxed in oxygenated conditions, and this may reflect the DNA supercoiling dynamics that occur as *S. enterica* approaches the aerobic region immediately adjacent to the intestinal epithelium [20], [21]. In tissue culture, *S. enterica* DNA supercoiling appears to remain static in epithelial cells but is dynamic when the bacterium resides inside macrophage [9], which demonstrates the complexity of *S. enterica*'s interactions with host environments. Our investigation of the links between environment, DNA supercoiling, and gene expression has uncovered a fundamental mechanism of SPI-1 and SPI-2 regulation in which relaxation of DNA supercoiling recruits OmpR to the *ssrA*, *hilC*, and *hilD* promoters, and this level of control functions independently of the fine-tuning effected by SPI-encoded transcription factors.

## Results

### Induction of SPI genes by DNA relaxation requires specific transcription factors

The *ssrAB* promoter (P*_ssrA_*) is induced by novobiocin, an aminocoumarin antibiotic that specifically inhibits the DNA supercoiling activity of the DNA gyrase subunit B (GyrB) (Figure 1B). In contrast, the SPI-2 T3SS and effector gene promoters, *ssaB-E*, *sseA-G*, *ssaG-L*, and *ssaM-R*, are only very slightly induced by altered DNA topology and the presumed increase in SsrA and SsrB concentrations brought about by novobiocin treatment (Figure S1A). Thus, the ability of DNA relaxation to activate SPI-2 is channeled through the cognate SsrA/B two-component regulator. P*_ssrA_* induction was reduced in cells lacking FIS, a master regulator of DNA supercoiling (Figure 1B), possibly because novobiocin has a reduced effect on DNA supercoiling in Δ*fis* mutants compared to wild type cells [20]. Unlike FIS, OmpR and its phospo-donor EnvZ were both absolutely required for induction of P*_ssrA_*, suggesting that relaxed DNA supercoiling alone cannot activate P*_ssrA_* in the absence of OmpR's ability to recruit RNAP. The requirement for EnvZ indicates that OmpR must be phosphorylated in order to stimulate these promoters, and also indicates that other phospho-donors do not activate OmpR in these conditions.

The SPI-encoded regulators HilD and SsrA/B played no detectable role in P*_ssrA_* induction. The alternate sigma factor RpoS is better at transcribing relaxed DNA than is the primary housekeeping sigma factor RpoD [22], and the elevated level of RpoS during stationary phase correlates with *ssrA* expression in standard laboratory conditions, but deletion of *rpoS* did not reduce P*_ssrA_* induction by novobiocin (Figure S1B).

Previous experiments in *S. enterica* have shown that the *ompR-envZ* promoter (P*_ompR_*) is induced by high concentrations of novobiocin at late stages of growth, and that OmpR is an auto-regulator of this induction [23]. We found that P*_ompR_* is also activated by low concentrations of novobiocin during exponential growth, and FIS and OmpR-EnvZ contribute to this induction (Figure 1B). Deletion of *hilD* did not affect P*_ompR_* induction. However, P*_ompR_* activity was unexpectedly elevated in the Δ*ssrA/B* mutant, suggesting that SsrA/B may directly or indirectly regulate *ompR* expression.

Because SPI-1 and SPI-2 genes are usually observed to have inverse expression patterns, we expected SPI-1 genes to be insensitive or repressed by DNA relaxation. We tested the effects of novobiocin treatment on expression of the master regulators *hilA*, *hilC*, and *hilD*, and were surprised to find that both the *hilC* and *hilD* promoters (P*_hilC_* and P*_hilD_*) were induced by DNA relaxation (Figure 1B). P*_hilA_* was insensitive to DNA relaxation (Figure S1A), suggesting that the inducing signal is limited to P*_hilC_* and P*_hilD_* (Figure 1A). Like P*_ssrA_*, P*_hilC_* required both FIS and OmpR-EnvZ for induction; yet unlike P*_ssrA_*, the absence of FIS was not compensated by increasing concentrations of novobiocin. Consistent with its role as a transcriptional activator, HilD was required for full activation of P*_hilC_* (Figure 1B). SsrA/B did not contribute to P*_hilC_* induction. P*_hilD_* was unique among the four promoters in having higher expression in the absence of *ompR* and *envZ*, but it nevertheless required FIS for full activation (Figure 1B). P*_hilD_* induction was unaffected by the absence of SsrA/B or HilD. Quantitative PCR measurement of *ssrA*, *ompR*, *hilC*, *hilD*, and *hilA* mRNA levels confirmed the results obtained from the reporter gene fusions (Figure S1C).

### OmpR requires DNA relaxation to stimulate transcription

**Figure 2:**
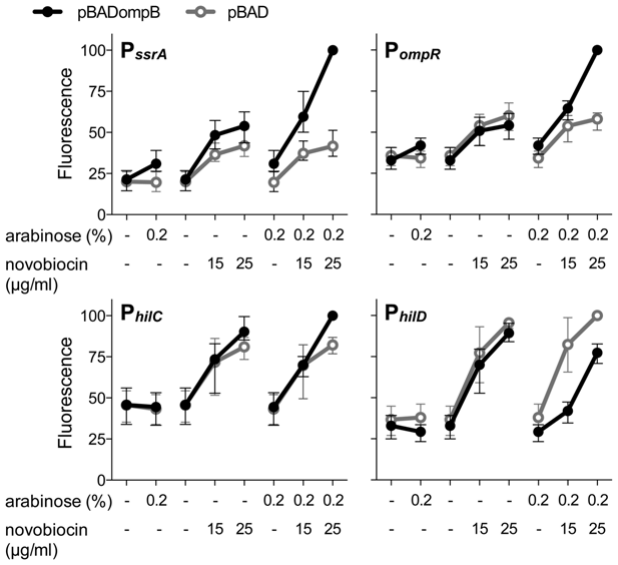
The relative contributions of OmpR and DNA relaxation to promoter activity. GFP production from transcriptional reporter fusions was measured in Δ*ompB* mutant cells carrying either an arabinose-inducible *ompR-envZ* operon (pBAD*ompB*) or the empty pBAD vector. The mean and range of expression from 3 biological replicates is plotted as in Figure 1B.

Having found that OmpR and relaxed DNA supercoiling work in concert to stimulate transcription from P*_ssrA_*, P*_hilC_*, and P*_ompR_*, we wished to test the relative contributions of OmpR and DNA topology to promoter function. To this end, the *ompR-envZ* operon (*ompB*) was cloned under the control of the arabinose-inducible P_BAD_ promoter in a Δ*ompB* mutant. P*_ssrA_* expression increased only very slightly when *ompB* was overexpressed (0.2% arabinose) in the absence of DNA relaxation (Figure 2). In contrast, DNA relaxation in the complete absence of OmpR (empty pBAD vector) had a stimulatory effect on P*_ssrA_*; this activation was higher in cells carrying pBADompB, likely due to leaky transcription of *ompB* in the absence of arabinose. The combination of *ompB* over-expression and DNA relaxation had the strongest stimulatory effect, confirming that OmpR and DNA relaxation work in concert to activate P*_ssrA_*. The combination of *ompB* over-expression and DNA relaxation also resulted in maximal expression of P*_hilC_* and P*_ompR_*, however the effect was more subtle for P*_hilC_* (Figure 2). Consistent with the results presented in Figure 1B, P*_hilD_* was repressed by *ompB* expression, and this repression occurred in both the absence and presence of DNA relaxation. Repression of P*_hilD_* was strongest at the lower concentration of novobiocin (15 µg/ml), raising the possibility that a high degree of DNA relaxation reduces repression by OmpR, perhaps through elevated HilC levels brought about by DNA relaxation (Figure 1A).

### OmpR binding to SPI-1 promoters

**Figure 3:**
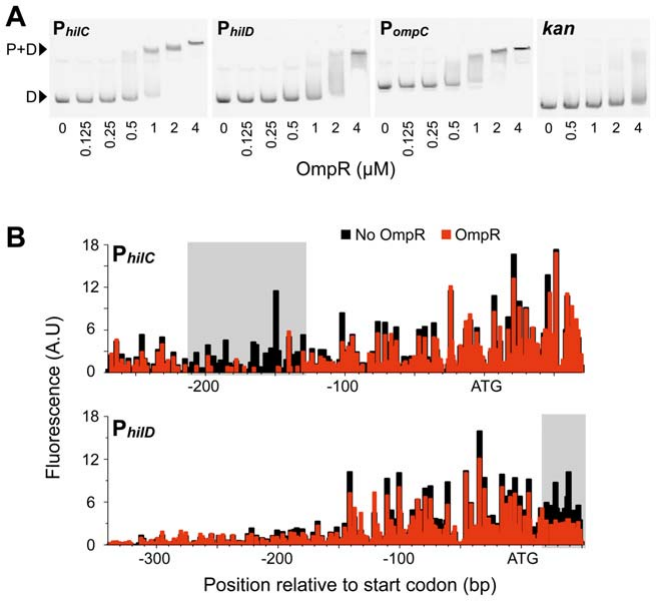
OmpR binding to SPI-1 gene promoters. A) Bandshifts showing OmpR binding to P*_hilC_* and P*_hilD_*, as well as to the P*_ompC_* positive control and the *kan* gene negative control. D, free DNA; P+D, protein-DNA complexes. B) Footprinting of OmpR binding to P*_hilC_* and P*_hilD_* using end-labelled linear DNA fragments. The size and quantity of 6-FAM-labelled digestion products were measured using a capillary electrophoresis DNA sequencing instrument.

The control of *hilC* and *hilD* expression by OmpR suggested that OmpR may regulate these genes through direct interactions. Electrophoretic mobility shift (bandshift) assays confirmed that OmpR binds specifically to both P*_hilC_* and P*_hilD_*, with OmpR demonstrating an affinity for P*_hilC_* similar to that for the positive control P*_ompC_* (Figure 3A). A negative control bait DNA (*kan*) was not bound specifically by OmpR at the concentrations tested. In these equilibrium binding assays, the rapid appearance of OmpR-DNA complexes over a small range of protein concentrations was evidence of cooperative OmpR binding to the bait DNA. Moreover, OmpR-DNA complexes demonstrated slower migration at higher OmpR concentrations, indicating that multiple OmpR molecules were bound to a single bait DNA molecule. Cooperative DNA binding is a feature common among NAPs— like FIS and H-NS— that bind with low-specificity to multiple proximal DNA sites [24], [25]. Indeed, OmpR monomers are thought to first bind cooperatively to form a nucleating dimer that recruits additional OmpR dimers in a cooperative fashion [26].

DNase footprinting revealed that P*_hilC_* and P*_hilD_* each have a single region protected by OmpR (Figure 3B). The P*_hilC_* region bound by OmpR is located over 100 bp upstream of the *hilC* start codon, consistent with OmpR's function as a transcriptional activator of this promoter. Conversely, the OmpR-protected region of P*_hilD_* is downstream of the *hilD* start codon, where OmpR binding is likely to have a repressive effect on *hilD* transcription.

### Promoter occupancy

If OmpR function is enhanced by relaxation of DNA supercoiling, does DNA relaxation result in increased DNA binding by OmpR? This was tested by quantifying OmpR binding to gene promoters *in vivo* using chromatin immuno-precipitation (ChIP). A fusion of the 3×-Flag epitope tag to the C-terminus of OmpR was used for these experiments. The epitope tag added 22 amino acids adjacent to OmpR's DNA-binding domain and created a new ribosome binding site for the *envZ* open reading frame; nonetheless, cells with OmpR:Flag showed only a slight reduction in promoter activation by novobiocin (Figure S1C). Alternatively, a 3×Flag tag at the N-terminus of OmpR could not be used because it generated a Δ*ompR* phenotype at promoters (not shown).

**Figure 4:**
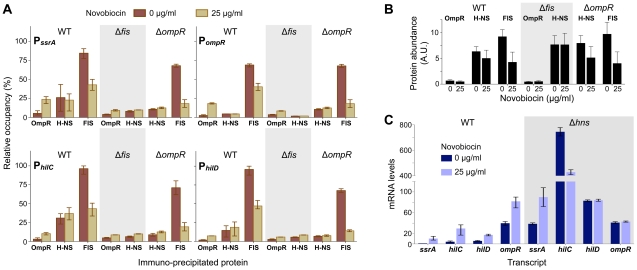
OmpR, FIS, and H-NS binding to promoter DNA. A) Quantification of protein binding to promoter DNA by immuno-precipitation 40 minutes after addition of novobiocin. The quantity of precipitated DNA is expressed as a percentage of maximal precipitation of the P*_hilC_* locus by FIS in wild type cells. The mean and range from 3 biological replicates is plotted. B) Protein levels in wild type and mutant cells 40 minutes after addition of novobiocin. Protein abundance was quantified by western blot analysis; the mean and standard deviation of three biological replicates is plotted. C) Quantitative PCR measurement of gene transcript levels in wild type and Δ*hns* mutant cells before and 40 minutes after addition of novobiocin. The mean and range of mRNA levels (expressed relative to *ssrA* in wild type cells at 0 µg/ml novobiocin) in three (wild type) and two (Δ*hns*) biological replicates are plotted.

Novobiocin treatment caused a significant increase in OmpR occupancy at P*_ssrA_*, P*_ompR_*, P*_hilC_*, and P*_hilD_* (Figure 4A). Increased promoter occupancy was due solely to a change in binding activity as OmpR levels were observed to decrease after novobiocin treatment (Figure 4B). Because OmpR requires DNA relaxation for it to be fully active at SPI promoters (Figure 2), we predicted that OmpR is an ineffective antagonist of H-NS binding and thus requires novobiocin-induced changes in DNA topology to assist in H-NS displacement. Our H-NS ChIP results confirm earlier studies that have found H-NS to occupy SPI promoters, but demonstrates a low affinity for P*_ompR_* [5], [27] (Figure 4A). Surprisingly, at all four promoters H-NS abundance was not affected by DNA relaxation nor by increased OmpR binding. It is important to note however that while ChIP quantifies protein abundance at genomic regions at a resolution around 500 bp, ChIP does not resolve changes to higher-order protein complexes if protein abundance remains constant. Therefore, we cannot rule out that although H-NS is not displaced, promoter activity may increase because H-NS oligomers are restructured by OmpR binding as well as by changes in DNA topology.

Chromatin immuno-precipitation of FIS fused to a 8×Myc epitope tag has been used previously to examine genome-wide FIS binding in *E. coli* [28]. We constructed an identical FIS:Myc fusion protein in *S. enterica* and used this to measure FIS occupancy of gene promoters in our experimental conditions. This revealed a high abundance of FIS at the SPI promoters, with slightly less FIS bound to P*_ompR_* (Figure 4A). At all loci tested, FIS occupancy decreased when cells were treated with novobiocin. This reduced FIS occupancy can be explained mostly by the ∼50% decrease in FIS levels in novobiocin-treated cells (Figure 4B). Although FIS contributes to transcriptional activation of these promoters, the finding that transcriptional activation occurs even when FIS is depleted suggests that FIS may act in part through its global control of DNA topology. Because FIS transitions from a filamentous DNA-binding mode to an ordered dimer as its concentration decreases [24], it is also possible that the depletion of FIS coupled with changes in DNA topology restructures FIS complexes into forms that favour transcription activation.

We next tested whether the decrease in promoter activity observed in a Δ*fis* mutant (Figure 1B) was due to a reduced ability by OmpR to access gene promoters. The ChIP data suggest that both OmpR and H-NS have less access to promoter DNA in a Δ*fis* mutant (Figure 4A). We have previously found that the Δ*fis* mutant is resistant to relaxation of DNA supercoiling by novobiocin [20]. It may be that H-NS and OmpR require DNA relaxation to gain full access to P*_ssrA_*, P*_ompR_*, P*_hilC_*, and P*_hilD_*, and the degree of relaxation is too modest in the Δ*fis* mutant. Nevertheless, novobiocin treatment caused a small increase in OmpR occupancy in Δ*fis* mutants, indicating that OmpR binding does not absolutely require the topological constraints imposed on DNA by FIS binding.

The same experiment was conducted in a Δ*ompR* mutant. In the absence of OmpR, novobiocin treatment caused a reduction in FIS binding (Figure 4A), again consistent with a reduction in FIS levels in these cells (Figure 4B). Although less H-NS bound to SPI promoters in the Δ*ompR* mutant, significantly more H-NS bound to P*_ompR_*, suggesting that OmpR is an effective H-NS antagonist at its own promoter. Surprisingly, the reduced H-NS levels observed in the Δ*ompR* mutant (Figure 4B), along with the further reduction in H-NS levels upon novobiocin treatment, implicates OmpR as a regulator of *hns* expression.

### Regulation in a Δ*hns* mutant

Because DNA relaxation does not appear to displace H-NS from gene promoters, we tested how removing H-NS from the system affects promoter function. Although all four test promoters had a similar pattern of induction by DNA relaxation in wild type cells, contrasting responses were observed in the absence of H-NS. As expected, all three SPI promoters were strongly upregulated (20 to 200-fold) in the Δ*hns* mutant (Figure 4C). In the absence of H-NS, *ssrA* was induced, *hilC* was repressed, and *hilD* was unaffected by DNA relaxation. These contrasting responses may result from the different and complex regulatory inputs acting at each promoter, and further confirm that promoter induction by DNA relaxation is not due simply to antagonism of H-NS repression.

Transcriptional output from P*_ompR_* was the same in wild type and Δ*hns* mutant cells in normal growth conditions (Figure 4C). This finding that H-NS does not repress P*_ompR_* is consistent with the low affinity of H-NS for this promoter (Figure 4A). Surprisingly though, P*_ompR_* was not induced by novobiocin in the Δ*hns* mutant, which may be indirectly caused by the highly pleiotropic effects of the Δ*hns* mutation.

### OmpR constrains a supercoiled-like state

**Figure 5:**
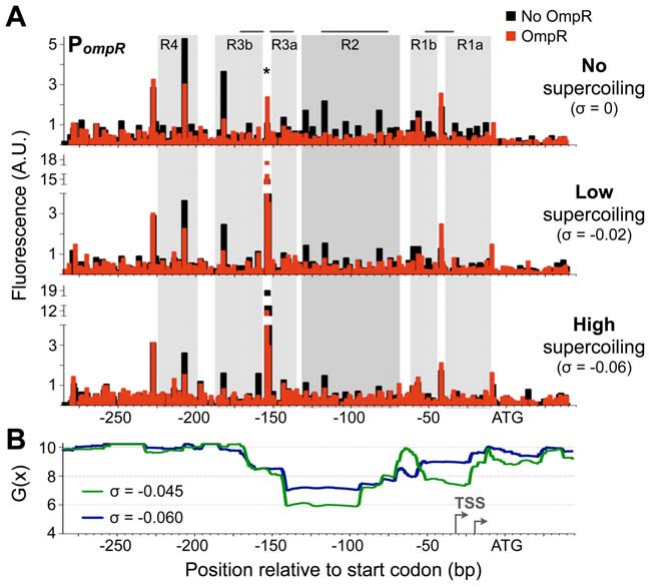
OmpR binding to DNA at different supercoiling levels. A) Quantification of OmpR binding to P*_ompR_* in the plasmid pZec-PompR at various supercoiling states using primer extension DNase I footprinting. The size and quantity of 6-FAM-labelled primer extension products were measured using a capillary electrophoresis DNA sequencing instrument. The approximate superhelical density (σ) of DNA in binding reactions is indicated. Horizontal grey lines above the footprints show where Bang *et al.* [23] observed OmpR footprinting; note that they measured binding to a limited region of the promoter. Grey-filled background highlights OmpR-protected regions, with darker grey highlighting the OmpR-2 site. The asterisk indicates the hypersensitive site at position −154. B) SIDD profile of P*_ompR_* in pZec-PompR at two superhelical densities. To detect long range effects of DNA supercoiling, WebSIDD [32] uses a default 5 kbp window that slides by 500 bp, thus each base pair is considered 10 times and the G(x) is calculated by weighting the effects of proximal bases. G(x) is expressed in kcal/mol/bp. Grey arrows indicate transcription start sites (TSS), mapped in [23].

To determine how DNA supercoiling affects OmpR affinity for DNA, we used primer extension to resolve DNase footprints on supercoiled and linear DNA templates. This approach can also determine if OmpR binds to different target sites depending on DNA supercoiling state, thus P*_ompR_* was used as the target DNA in this set of experiments because it has multiple, clearly delineated OmpR binding sites [23]. The grey filled boxes in Figure 5A highlight the regions of P*_ompR_* protected from DNase I digestion by OmpR. Most protection in the absence of supercoiling (linear DNA) was observed at a 60 bp region, OmpR-2, with lesser protection of regions on either side. Protected regions were assigned numbers to correspond with the OmpR sites identified previously by Bang *et al.* [23] (horizontal, dark-grey lines). Unlike Bang *et al.* [23], we analyzed OmpR binding to the full P*_ompR_* intergenic region to resolve a more promoter-distal site, OmpR-4. DNase I digestion of an end-labeled linear template confirmed that OmpR-2 is the primary site of OmpR binding to P*_ompR_* (Figure S2A).

As supercoiling levels increased, protection by OmpR appeared to decrease (Figure 5A), giving the impression that OmpR binds DNA better at lower supercoiling levels. However, this result was caused by a decrease in DNase I cutting of unprotected supercoiled DNA, perhaps due to DNA compaction and the loss of B-DNA conformation at higher superhelical densities. The amount of DNase I digestion in the presence of OmpR was consistent regardless of the superhelical density of P*_ompR_* DNA. Thus, OmpR may reduce DNase I digestion across the entire promoter region by constraining a supercoiled-like state in DNA, as has been observed for FIS and H-NS [29], [30].

DNA positions that are hypersensitive to endonuclease cutting offer additional insight into changes in DNA topology. For example, positions −207 and −182 became less sensitive whereas positions −154 and −9 became increasingly sensitive to DNase I digestion as supercoiling increased. Position −154 (marked with an asterisk) is particularly intriguing because it was ultra-hypersensitive to DNase I digestion when DNA was supercoiled. When DNA was fully relaxed, OmpR binding greatly enhanced DNase I cutting at position −154, supporting a model in which OmpR binding creates DNA structures similar to those induced by negative DNA supercoiling.

DNA supercoiling exerts torsional stress that weakens base pairing, and so reduces the amount of energy needed for DNA melting and transcription initiation. This is referred to as stress-induced duplex destabilization (SIDD), and the energy required for strand separation at each base pair in a specific sequence, G(x), can be predicted for different superhelical densities [31]. Stable base pairs have G(x) values around 10, whereas lower values indicate positions prone to SIDD. We used WebSIDD [32] to predict the stability of P*_ompR_* DNA at the approximate superhelical densities observed during exponential growth (σ = −0.06) and after treatment with 15 µg/ml novobiocin (σ = −0.045). The G(x) profiles of P*_ompR_* at both superhelical densities revealed a highly destabilized region ranging from positions −70 to −160, with a weakly destabilized region (−20 to −55) encompassing the *ompR* transcription start sites (Figure 5B). This analysis makes the counterintuitive prediction that P*_ompR_* becomes increasingly destabilized as DNA relaxes, which is nevertheless consistent with the observed gene activation in these conditions (Figure 1B). DNA upstream of position −170 is highly stable, indicating that the destabilization effect is specific and concentrated at the main binding site used by OmpR. The primary OmpR binding sites, OmpR-1 and OmpR-2, cover most of the highly destabilized regions, raising the possibility that OmpR binding transmits the destabilizing force to the adjacent RNA polymerase binding site where DNA strand separation can assist in transcription initiation. A similar SIDD-transmission function has been characterized for FIS and IHF [33].

Novobiocin treatment caused the same degree of DNA relaxation in Δ*ompR* mutant cells as in wild type cells (Figure S2B). This suggests that promoter DNA experiences the same stress-induced strand destabilization in both mutant and wild type, indicating that reduced promoter activation in the Δ*ompR* mutant (Figure 1B) is due to the absence of OmpR binding, not an altered degree of DNA relaxation. In other words, DNA strand destabilization caused by DNA supercoiling is insufficient for P*_ompR_* activation in the absence of OmpR binding.

## Discussion

*S. enterica* traverses various extracellular and intracellular environments during infection of host tissues, thus it requires genetic programs capable of balancing shifting requirements for the T3SS and effector proteins that mediate the invasion process. Two recent studies unexpectedly discovered that SPI-2 genes are expressed in the mouse intestinal lumen prior to cellular invasion, leading to the hypotheses that SPI-2 is either important for colonization of the intestine or requires priming before intracellular invasion [12], [34]. SPI-2 expression during growth in rich medium, which roughly mimics conditions in the intestinal lumen, requires OmpR and FIS but is independent of SsrB, SlyA, and PhoP [12], [18]. Here we describe a fundamental mechanism that activates both SPI-2 and SPI-1 promoters through changes in DNA topology, and this mechanism depends on OmpR and FIS but is independent of SsrB and HilD. It is intriguing that P*_ssrA_* induction does not require FIS in culture conditions that mimic the vacuolar environment [12], nor is FIS required for P*_ssrA_* induction when DNA is highly relaxed [20]. These findings support a model in which fine-tuning of SPI gene expression by factors such as SsrB, HilD, SlyA, and PhoP may occur primarily in the vacuolar environment.

Although OmpR-EnvZ is the archetypal two component signal transduction pathway, the environmental stimulus of EnvZ kinase activity remains unclear [35], and this stimulus appears to differ between *E. coli* and *S. enterica* [36]. It is perhaps for this reason that a role for OmpR in the regulation of SPI-1 has been enigmatic. Previous studies have found no effect or only weak effects from deletion of *ompR* or *envZ*. It was initially proposed that OmpR-EnvZ directly controls *hilC* expression [37], but others have favoured a model in which OmpR somehow acts post-transcriptionally through HilD protein function [2]. Here we provide evidence that under conditions of relaxed DNA supercoiling, OmpR binds directly to both the *hilC* and *hilD* promoters where it activates the former and represses the latter.

There is a growing body of evidence that in addition to the classic role of OmpR as a site-specific transcription factor that activates gene expression through RNAP recruitment, it also exhibits NAP-like features and functions. Because OmpR makes few specific contacts with DNA, it demonstrates an affinity for non-specific DNA [38], [39]. The preferred OmpR target sites, which are highly degenerate at the sequence level, may serve as nucleating points for cooperative recruitment of additional OmpR molecules. Nucleation and cooperative DNA binding can explain the broad regions of OmpR protection observed at P*_hilC_* (Figure 3B), P*_ompR_* (Figure 5A), and P*_ssrA_* [10]. Additionally, our ChIP data revealed OmpR binding to regions not predicted to be specific targets (Figure S2C). This is similar to the ChIP survey of cAMP receptor protein (CRP) targets in *E. coli* which revealed a high background of CRP binding across the entire chromosome [40]. CRP binding to thousands of weak sites lead the authors to propose that this archetypal transcription factor should also be considered as a chromosome-structuring protein. An additional interesting parallel between OmpR and CRP is that both are calculated to have similar cellular concentrations: ∼3,000 CRP and ∼3,500 OmpR molecules/cell [41], [42]. Thus, both OmpR and CRP may represent a class of NAP-like DNA-binding proteins whose high abundance allows them to have a broad influence on chromosome shape and function, yet whose modulation through allosteric effectors generates titratable DNA-binding modes that preferentially target specific promoters.

The discovery that DNA relaxation results in increased OmpR binding to DNA *in vivo* presents an intriguing model in which this mechanism is complementary to phosphorylation of OmpR by EnvZ as a means to stimulate OmpR-DNA binding. Thus, phosphorylated OmpR may be recruited to promoters by DNA relaxation. Future global analysis of OmpR binding to the *S. enterica* chromosome will shed light on the relative contributions of phosphorylation and DNA relaxation to OmpR-DNA interactions.

Whole-genome analysis of the transcriptional consequences of DNA relaxation in *E. coli* revealed that relaxation-induced promoters are significantly more A+T-rich than are uninduced promoters [43]. Because H-NS preferentially binds to regions of high A+T content, relaxation-induced promoters are very likely to be H-NS repressed. In *E. coli*, the OmpR targets P*_ompR_* and P*_ompC_* were induced whereas P*_ompF_* was repressed by novobiocin [43], and we found the same response in *S. enterica* (Figure S2D). This shared response of OmpR-regulated genes to novobiocin in *E. coli* and *S. enterica* coupled with the proposed ability of DNA relaxation to weaken H-NS repression hints at an evolutionarily conserved gene regulatory mechanism that predates horizontal acquisition of SPI-1 and SPI-2 by *Salmonella*.

Transcriptional activators of SPI genes (HilC, HilD, SsrB, and SlyA) function in large part through displacement of H-NS from SPI promoters [16], [44]. Members of the AraC-like protein family, which includes HilC and HilD, have a well-documented ability to displace H-NS [45]. SsrB, a member of the NarL protein family, activates transcription by displacing H-NS but does not appear able to break H-NS bridges [44]. OmpR and SlyA are winged-helix DNA binding proteins. Like OmpR, SlyA relieves H-NS repression without displacing H-NS [46]; SlyA also generates regions of DNase I hypersensitivity, thus may have a topological restructuring mode that contributes to breaking H-NS bridges [46]. However, unlike OmpR, SlyA relies on activators such as PhoP to recruit RNAP. Variable modes of H-NS antagonism — from anti-polymerization by HilD, HilC, and SsrB to anti-bridging by OmpR and SlyA — may represent a gate-keeper mechanism that selects which of the numerous regulators known to act at SPI gene promoters are allowed access to their target DNA sites, thus fine-tuning transcriptional output.

## Materials and Methods

### Strains

*Salmonella enterica* serovar Typhimurium strain SL1344 was used for all experiments. Detailed descriptions of mutant strains used in this study are provided in Table S1. *E. coli* XL-1 blue was used for all cloning steps.

### Mutant construction

Strains and plasmids used in this study are listed in Table S1. To generate *S. enterica* mutants, the kanamycin resistance cassette was PCR amplified from pKD4 [47] using primers listed in Table S2, which were designed to replace only open reading frames. Because the *ompR* and *envZ* open reading frames overlap, special care was taken to preserve open reading frames when constructing deletion and epitope-fusion mutations.

PCR amplicons were spin column purified then transformed into electrocompetent *S. enterica* SL1344 containing the Red helper plasmid pKD46 as previously described [47], [48]. Mutations were transduced into a fresh SL1344 background by bacteriophage P22 generalized transduction [49], then were confirmed by DNA sequencing.

Site-directed mutagenesis of the *ompB* locus cloned in pUC18 was carried out using the QuikChange II kit (Stratagene) and primers listed in Table S2, following the manufacturer's protocol.

### Cloning

Transcriptional reporter fusions were constructed by cloning gene promoters in pZep and pZec vectors, which contain a promoterless *gfp*+ gene [20], [50]. The *cat* gene was removed from pZep to generate pZec so that transcriptional reporter fusions could be cloned in cells containing pBAD. The removal of the *cat* gene from pZep to generate pZec had no effect on expression of cloned promoters (not shown). Data presented in Figure 1 and Figure 2 is from cells carrying pZec reporter plasmids.

pZep or and pZec plasmids were digested with SmaI and XbaI, spin column purified using the HiYield PCR DNA Fragment Extraction Kit (RBC Bioscience), and dephosphorylated with Antarctic Phosphatase (New England Biolabs). Gene promoter sequences were PCR amplified using the Phusion DNA polymerase (NEB) and primers listed in Table S2. SmaI and XbaI digested amplicons were spin column purified, then ligated to pZep or pZec by T4 ligase (Roche). The exception was the *ompR* promoter region which was digested with NotI and XbaI before cloning into similarly digested vector.

The *ompB* locus along with its native ribosome binding site was PCR amplified using primers listed in Table S2, followed by PstI and SacI digestion and spin column purification. PstI and SacI digested pBAD33 was gel purified, de-phosphorylated by Antarctic Phosphatase (NEB), then ligated to the digested PCR amplicon. The effects of inducible *ompB* on promoter function were assessed by measuring GFP levels in cells carrying both pBAD*ompB* and pZec transcriptional reporter clones. Arabinose and novobiocin were added to cultures at the concentrations indicated in Figure 2.

### Culture conditions and novobiocin treatment

Cells were cultured in a shaking waterbath at 37°C in LB (1% tryptone and 0.5% yeast extract) without any NaCl added. Cells used for *gfp* reporter fusion experiments were cultured in 4 ml of LB in glass tubes (interior diameter 14 mm) shaking at 200 RPM whereas cells used for ChIP and quantitative PCR experiments were cultured in 55 ml of LB in 250 ml glass flasks shaking at 140 RPM. Previous studies testing the effects of novobiocin on gene expression in *S. enterica* have used high concentrations of novobiocin (25–150 µg/ml) in cells transitioning from late exponential to stationary phase physiology [9], [23], thus introducing additional variables arising from growth phase transitions. To ensure a steady state of growth, we conducted all experiments using cells that had been growing exponentially for more than six doublings at low cell density (OD_600_ less than 0.3). In addition, we used low concentrations of novobiocin (15–25 µg/ml) to minimize effects on growth rate. Cells were fixed after 3 hrs of continued growth at low density in the presence or absence of novobiocin (final OD_600_ 0.1–0.25), as in [20].

### Quantitative PCR measurement of gene expression

Total RNA was isolated from cultures using the SV Total RNA Isolation System (Promega) and purity and quality was assessed by electrophoresis in 1% agarose (1×TAE). For each sample, 5 µg total RNA was DNase treated in a 50-µl reaction using the Turbo DNA-free kit (AMBION), and cDNA templates were synthesized by random priming 0.5 µg RNA in a 20 µl reaction using the GoScrip Reverse Transcription System (Promega). Quantitative PCR (qPCR) primers are listed in Table S2. PCR reactions were carried out in duplicate with each primer set on an ABI 7500 Sequence Detection System (Applied Biosystems) using FastStart SYBR Green Master with ROX (Roche). Standard curves were included in every qPCR run; standard curves were generated for each primer set using five serial tenfold dilutions of *S. enterica* chromosomal DNA.

### Chromatin immuno-precipitation (ChIP)

ChIP was conducted as previously described [27]. Two ChIP replicates were performed using a strain containing both the *ompR:flag* and *fis:myc* epitope fusions, allowing for simultaneous precipitation of OmpR:Flag, Fis:Myc, and H-NS from the same biological sample. One ChIP replicate was conducted for each strain carrying a single epitope tag (*ompR:flag* or *fis:myc*). ChIP results overlapped between the double and single fusion strains, indicating that the epitope-tagged proteins did not negatively affect nucleoprotein interactions when combined. Precipitated DNA was quantified by quantitative PCR using primers listed in Table S2.

### Western blot analysis

Cells were pelleted and resuspended in 1× Laemmli buffer (4% SDS, 20% glycerol, 10% 2-mercaptoethanol, 0.004% bromophenol blue, 0.125 M Tris HCl, pH 6.8) and denatured at 100°C for 5 min. Samples were electrophoresed on 15% polyacrylamide SDS gels. Gels and nitrocellulose membranes were equilibrated in transfer buffer (25 mM Tris HCl, 192 mM glycine, 0.02% SDS, 20% methanol) and proteins were transferred to membranes at 150 V for 90 min using a Trans-blot (BioRad) apparatus packed in ice. Membranes were blocked overnight at 4°C in 5% non-fat powdered milk in PBS (137 mM NaCl, 12 mM Phosphate, 2.7 mM KCl, pH 7.4), followed by incubation at room temperature for 2 hr with rocking in primary antibodies diluted as follows: 1/100,000 anti-DnaK mAb rabbit (Enzo Life Sciences), 1/10,000 anti-FLAG mAb rabbit (Sigma), 1/10,000 anti-Myc mAb rabbit (Sigma), and 1/5,000 anti-H-NS polyclonal mouse [5]. Blots were washed thoroughly and probed with horseradish peroxidase-linked anti-rabbit and anti-mouse antibodies (Millipore) diluted 1/5,000 in PBS (1% blocking agent) for 1 hr at room temperature with rocking, followed by thorough washing. Blots were incubated in ECL reagent (Pierce) for 1 min, and bands were visualized using an ImageQuant LAS 4000 scanner (GE Healthcare) then quantified using ImageJ v1.43 (National Institutes of Health, U.S.A.). Probing for all proteins (DnaK, FIS:Myc, H-NS, and OmpR:Flag) simultaneously on the same blot allowed for protein quantities to be normalized to the internal standard (DnaK) and expressed relative to one another. Each cell sample was run on three independent western blots to improve the accuracy of quantification. Thus, the protein abundance value for each biological replicate is the average value from three replicate blots.

### OmpR purification

The OmpR D55E mutation creates a constitutively active protein by mimicking phosphorylation [51]. OmpR(D55E) with a C-terminal His-tag was purified and used in bandshifts and DNase I footprinting. BL21 cells carrying the pET21-*ompR(D55E)* plasmid were grown in L broth (0.5% NaCl; 100 µg/ml carbenicillin) and *ompR(D55E)* expression was induced at OD_600_ 0.5 with 1 mM IPTG. Cells were harvested after 4.5 hr by centrifugation and the pellet were frozen overnight at −20°. Native OmpR(D55E)-His was purified as follows: the pellet was resuspended in lysis buffer (50 mM sodium phosphate, 300 mM sodium chloride, 10 mM imidazole), then treated with 1 mg/ml lysozyme for 30 min at 24°C followed by sonication on ice. Insoluble material was removed by centrifugation at 10,000 g for 25 min and the supernatant was then incubated with nickel-nitriloacetic acid agarose beads for 1 hr at 4°C with gentle rocking. The agarose beads were loaded in a column and washed twice with four column volumes of wash buffer (50 mM NaH_2_PO_4_, 300 mM NaCl, 20 mM imidazole, pH 8.0), and protein was collected in elution buffer (50 mM NaH_2_PO_4_, 300 mM NaCl, 250 mM imidazole, pH 8.0). Purified protein was desalted with Nanosep 3K Omega membranes (Pall) at 4°C, then resuspended in storage buffer (20% glycerol, 40 mM Tris, 200 mM KCl) and stored at −80°. OmpR(D55E) purity was assessed on Coomassie stained SDS-PAGE gels, and concentration was quantified by both the Bradford assay and by comparison to protein standards on Coomassie stained SDS-PAGE gels.

### Bandshifts

Bait DNA was PCR amplified using the primers pZec.6FAM.R (labeled with a 5′ 6-FAM fluorophore) and pZec.confirm.F (Table S2), from pZec promoter clones. Amplicons were spin column purified then used as bait DNA in bandshifts. OmpR-DNA binding reactions (10 µl) contained 0.2× TBE (89 mM Tris, 89 mM borate, 2 mM EDTA (pH 8.3), 40 µg/µl poly(dI-dC) DNA, and 40 nM bait DNA. Reactions were incubated at room temperature for 15 min before being loaded onto a running polyacrylamide gel (30∶1 acrylamide/bisacrylamide, 0.2× TBE, 2% glycerol) with 0.2× TBE running buffer. After electrophoresis for 40 min at 100 V, 6-FAM-labeled DNA was visualized using a Typhoon scanner (GE Healthcare).

### DNase I footprinting of OmpR binding to PCR-amplified gene promoters

Bait DNA was prepared as for bandshifts using either the primer sets pZec.6FAM.F and pZec.confirm.R (top strand) or pZec.6FAM.R and pZec.confirm.F (bottom strand). DNase I footprinting reactions were conducted in 15 µl reaction volumes containing 1× DNase I buffer (Roche)(40 mM Tris-HCl, 10 mM NaCl, 6 mM MgCl_2_, 1 mM CaCl_2_; pH 7.9), 0.01 mM dithiothreitol, 100 ng/µl BSA, 50 nM bait DNA, and 5 µM OmpR(D55E)-His. OmpR-DNA binding was allowed to equilibrate at 37°C for 15 minutes, then 1 µl (0.015 units) of pre-warmed DNase I was added and mixed gently, then incubated at 37°C for 10 minutes. Reactions were stopped by addition of 2 µl EDTA (100 mM) followed by vigorous vortex mixing and heat denaturation at 95°C for 10 min. Digestion products were desalted using MicroSpin G-50 columns (GE Healthcare) and were analyzed on an ABI 3130 Genetic Analyzer along with GeneScan 500-LIZ size standards (Applied Biosystems).

### DNase I footprinting of OmpR binding to supercoiled gene promoters

Plasmids with varying degrees of superhelical density were generated as follows: pZec-PompR was purified from *E. coli* CSH50 at different topological states by growing cells overnight in 25 ml L (0% NaCl) in a well-aerated 250 ml glass flask (low supercoiling) or overnight in 6 ml L (0.5% NaCl) in 14 mm diameter glass culture tubes (high supercoiling). Topoisomers at the desired topological state were purified after separation on a 1% agarose gel containing 2.5 µg/ml chloroquine. The average superhelical density of each purified plasmid pool was determined by calculating the linking difference between the dominant topoisomer and fully relaxed DNA [52]. A 1% agarose gel containing 25 µg/ml chloroquine was used to improve the resolution of topoisomers in the low supercoiling sample; in these conditions, more relaxed DNA migrates faster through the gel. To generate a plasmid pool that lacked supercoiling, pZec-ompR was digested with XhoI, which cuts 500 bp away from the cloned promoter.

Because nicking of plasmid DNA by DNase I will allow DNA supercoils to relax, footprinting reactions were treated with DNase I for no more than 1 minute to reduce the time in which nicked plasmids could lose their topology. In these reactions, 1 µl (0.15 units) of DNase I was added to 15 µl OmpR-DNA binding reactions containing 10 µM OmpR and 1.5 nM bait DNA. OmpR-DNA binding was equilibrated as above.

Primer extension was conducted using the primer pZec.6FAM.F and Thermo Sequenase polymerase (USB) with the following thermocycle: 95°C for 30 sec, 53°C for 30 sec, and 72°C for 90 sec, repeated 50 times. Extension reactions contained 0.4 nM of nicked plasmid template. Amplification products were desalted using MicroSpin G-50 columns (GE Healthcare) and were analyzed on an ABI 3130 Genetic Analyzer along with GeneScan 500-LIZ size standards (Applied Biosystems). To compare samples, each was normalized to have the same total fluorescence signal across the DNA region being analysed.

## Supporting Information

Figure S1 Transcriptional control of SPI-1 and SPI-2 gene expression. A) Expression of SPI-2 and SPI-1 transcriptional reporter fusions in response to novobiocin. Fluorescence values are percentages relative to P*_ssrA_*:*gfp* at 25 µg/ml novobiocin. The mean and standard deviation of 3 biological replicates are plotted. B) Expression of P*_ssrA_*:*gfp* in wild type and Δ*rpoS* genetic backgrounds. Values indicate the percentage of fluorescence relative to wild type cells at 25 µg/ml novobiocin. C) Quantitative PCR measurement of gene transcript levels in wild type and *ompR:flag* cells before and 40 minutes after addition of novobiocin. The mean and standard deviation of mRNA levels (expressed relative to *ssrA* in wild type cells at 0 µg/ml novobiocin) in three (wild type) and two (*ompR:flag*) biological replicates are plotted. All transcripts were quantified relative to the same chromosomal DNA standard; *hilA* transcript levels were at the limit of accurate detection.
(TIF)

Figure S2 OmpR-DNA interactions. A) DNase I footprinting of OmpR binding to linear, end-labeled P*_ompR_* DNA. Grey shading highlights the OmpR binding regions observed in Figure 5A. B) DNA relaxation in response to subinhibitory concentrations of novobiocin (15 µg/ml). Two independent biological replicates are shown for each strain; pUC18 supercoiling reporter plasmids were prepared and analyzed in a 1% agarose gel containing 2.5 µg/ml chloroquine, as described in [20]. C) Quantification of OmpR:Flag binding to promoter DNA by immuno-precipitation 40 minutes after addition of novobiocin. The mean and range of enrichment values (arbitrary units) from 2 to 4 biological replicates are plotted. P*_hilA_*, P*_proU_*, P*_bamA_* are not known to be specific targets of OmpR. However, the *proU* locus is important for osmo-protection and *bamA* is essential for outer membrane protein biogenesis, thus both are plausible targets for OmpR regulation. The *guaC* open reading frame is not expected to be an OmpR target. D) Expression of P*_ompC_* and P*_ompF_* transcriptional reporter fusions in response to novobiocin. Values indicate the percentage of fluorescence relative to P*_ompC_*:*gfp* at 15 µg/ml novobiocin. The mean and standard deviation of 4 biological replicates are plotted.
(TIF)

Table S1 Bacterial strains used in this study. The table provides details of the strains of *Escherichia coli, Salmonella enterica* and plasmids used in the experiments described in the text. The sources of these materials or references to papers giving this information is also included.
(PDF)

Table S2 Oligonucleotide primers used in this study. The table reports the DNA sequences of primers used for cloning, quantitative PCR, mutant construction, DNase I footprinting or electrophoretic mobility shift assays (bandshifts).
(PDF)
