# Supplementary figures and images for: A Fundamental Regulatory Mechanism Operating through OmpR and DNA Topology Controls Expression of Salmonella Pathogenicity Islands SPI-1 and SPI-2
Source: PLoS Genet. 2012 Mar 22;8(3):e1002615. doi: 10.1371/journal.pgen.1002615 (PMC3310775; doi:10.1371/journal.pgen.1002615)

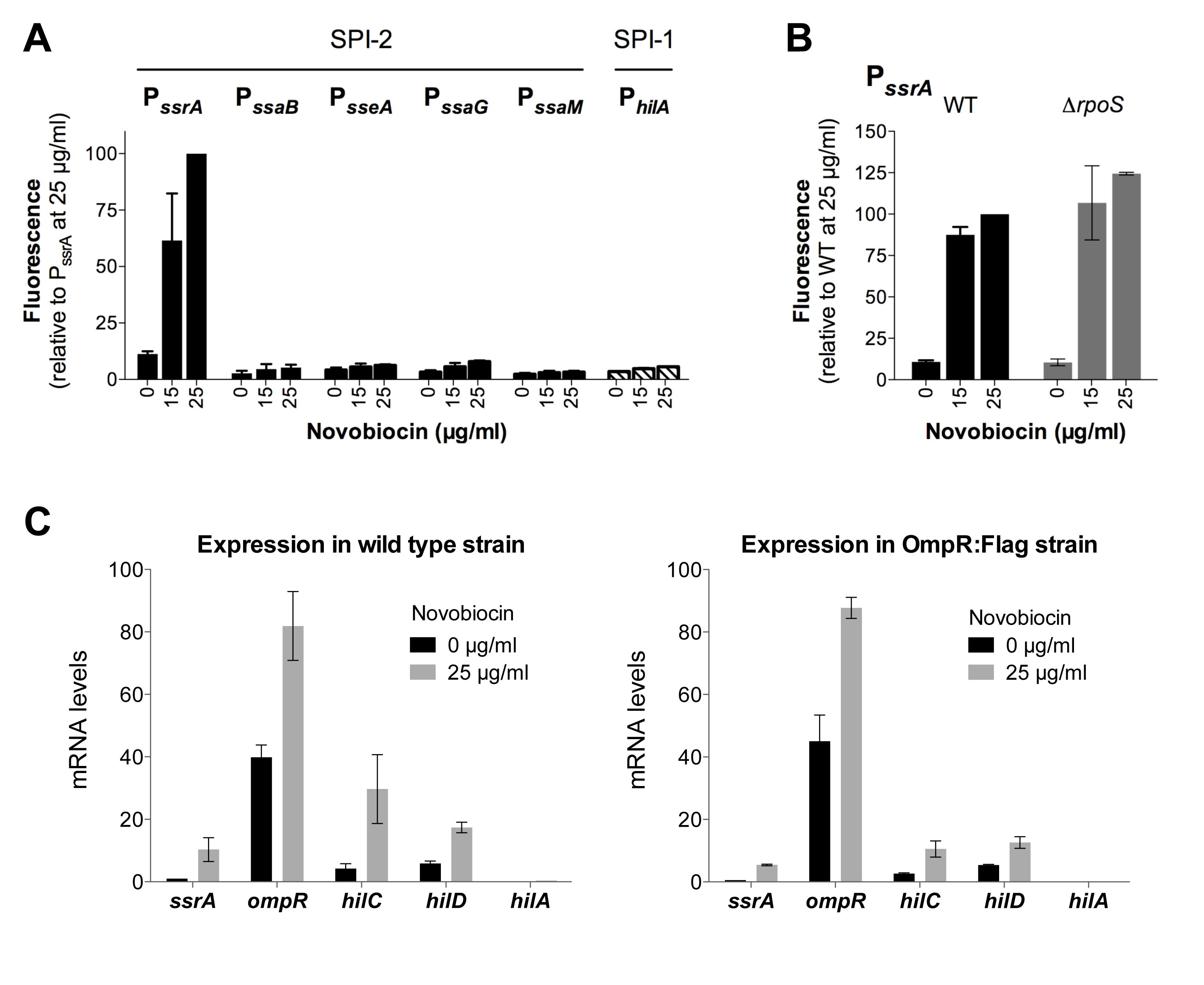

Supplement: Figure S1 — Transcriptional control of SPI-1 and SPI-2 gene expression. A) Expression of SPI-2 and SPI-1 transcriptional reporter fusions in response to novobiocin. Fluorescence values are percentages relative to PssrA:gfp at 25 µg/ml novobiocin. The mean and standard deviation of 3 biological replicates are plotted. B) Expression of PssrA:gfp in wild type and ΔrpoS genetic backgrounds. Values indicate the percentage of fluorescence relative to wild type cells at 25 µg/ml novobiocin. C) Quantitative PCR measurement of gene transcript levels in wild type and ompR:flag cells before and 40 minutes after addition of novobiocin. The mean and standard deviation of mRNA levels (expressed relative to ssrA in wild type cells at 0 µg/ml novobiocin) in three (wild type) and two (ompR:flag) biological replicates are plotted. All transcripts were quantified relative to the same chromosomal DNA standard; hilA transcript levels were at the limit of accurate detection. (TIF) [file pgen.1002615.s001.tif]

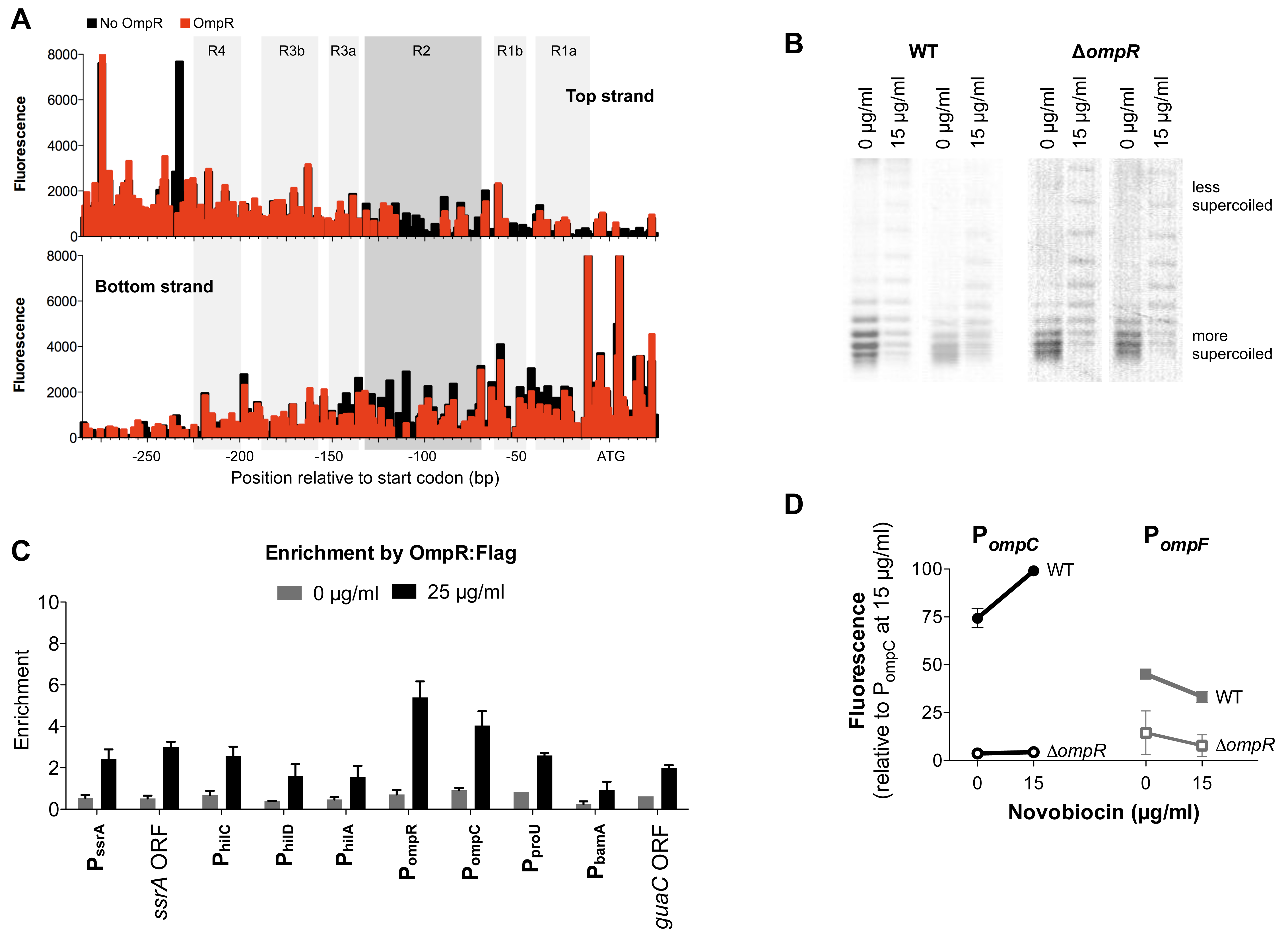

Supplement: Figure S2 — OmpR-DNA interactions. A) DNase I footprinting of OmpR binding to linear, end-labeled PompR DNA. Grey shading highlights the OmpR binding regions observed in Figure 5A. B) DNA relaxation in response to subinhibitory concentrations of novobiocin (15 µg/ml). Two independent biological replicates are shown for each strain; pUC18 supercoiling reporter plasmids were prepared and analyzed in a 1% agarose gel containing 2.5 µg/ml chloroquine, as described in [20]. C) Quantification of OmpR:Flag binding to promoter DNA by immuno-precipitation 40 minutes after addition of novobiocin. The mean and range of enrichment values (arbitrary units) from 2 to 4 biological replicates are plotted. PhilA, PproU, PbamA are not known to be specific targets of OmpR. However, the proU locus is important for osmo-protection and bamA is essential for outer membrane protein biogenesis, thus both are plausible targets for OmpR regulation. The guaC open reading frame is not expected to be an OmpR target. D) Expression of PompC and PompF transcriptional reporter fusions in response to novobiocin. Values indicate the percentage of fluorescence relative to PompC:gfp at 15 µg/ml novobiocin. The mean and standard deviation of 4 biological replicates are plotted. (TIF) [file pgen.1002615.s002.tif]
