# Supplementary material for: A Fundamental Regulatory Mechanism Operating through OmpR and DNA Topology Controls Expression of Salmonella Pathogenicity Islands SPI-1 and SPI-2
Source: PLoS Genet. 2012 Mar 22;8(3):e1002615. doi: 10.1371/journal.pgen.1002615 (PMC3310775; doi:10.1371/journal.pgen.1002615)
Supplement: Table S1 — Bacterial strains used in this study. The table provides details of the strains of Escherichia coli, Salmonella enterica and plasmids used in the experiments described in the text. The sources of these materials or references to papers giving this information is also included. (PDF) [file pgen.1002615.s003.pdf]

Supporting Table 1: Strains used in this study

Strain or plasmid	Description / genotype	Reference
E. coli		
XL-1	*recA1 endA1 gyrA96 thi-1 hsdR17 supE44 relA1 lac*	Stratagene
BL21	*E. coli* B, F- *dcm ompT hsdS(rB- mB-) gal*	[1]
CSH50	F- λ- *ara* Δ(*lac-pro*) *rpsL thi fimE*::IS*1*	[2]
***S. enterica* serovar Typhimurium**		
SL1344	*rpsL hisG*	[3]
SL1344*fis*::*kan*	*fis*, Kan^r	This study
SL1344*ompR::kan*	*ompR*, Kan^r	This study
SL344*envZ::kan*	*envZ*, Kan^r	This study
SL1344*ompB::kan*	*ompR-envZ*, Kan^r	This study
SL1344*ssrAB::kan*	*ssrAB*, Kan^r	This study
SL1344*hilD::kan*	*hilD*, Kan^r	[4]
SL1344*rpoS*::*kan*	*rpoS*, Kan^r	[5]
SL1344*ompR:flag*	*ompR:flag* epitope fusion	This study
SL1344*fis:myc*	*fis:myc* epitope fusion	This study
SL1344*ompR:flag/fis:myc*	*ompR:flag* and *fis:myc* epitope fusions	This study
**Plasmids**		
pBAD33	Cm^r	[6]
pBAD-*ompB*	pBAD33 containing *S. enterica ompR-envZ*	This study
pUC18	Amp^r (Carb^r)	
pZec	Amp^r (Carb^r), promoterless *gfp*+	[7]
pZec-PssrA	P_{ssrA} cloned in pZec	[7]
pZec-PhilC	P_{hilC} cloned in pZec	This study
pZec-PhilD	P_{hilD} cloned in pZec	This study
pZec-PompR	P_{ompR} cloned in pZec	This study
pZep	Amp^r (Carb^r), Cm^r, promoterless *gfp*+	[8]
pZep-PssrA	P_{ssrA} cloned in pZep	This study
pZep-PhilC	P_{hilC} cloned in pZep	This study
pZep-PhilD	P_{hilD} cloned in pZep	This study
pZep-PompR	P_{ompR} cloned in pZep	This study
pZep-PhilA	P_{hilA} cloned in pZep	This study
pZep-PssaB	P_{ssaB} cloned in pZep	This study
pZep-PsseA	P_{sseA} cloned in pZep	This study
pZep-PssaG	P_{ssaG} cloned in pZep	This study
pZep-PssaM	P_{ssaM} cloned in pZep	This study
pET21-ompR(D55E)	*ompR(D55E)* cloned in frame with C-terminal His-tag	This study
pBOP508	8xMyc epitope	[9]

Abbreviations: Amp^r, ampicillin resistance; Carb^r, carbenicillin resistance; Cm^r, chloramphenicol resistance; Kan^r, kanamycin resistance

References

1. Studier FW, Moffatt BA (1986) Use of bacteriophage T7 RNA polymerase to direct selective high-level expression of cloned genes. J Mol Biol 189: 113-130.
2. Miller JH (1972) Experiments in Molecular Genetics. Plainview, N.Y.: Cold Spring Harbor Laboratory Press.
3. Hoiseth SK, Stocker BA (1981) Aromatic-dependent *Salmonella typhimurium* are non-virulent and effective as live vaccines. Nature 291: 238-239.
4. Bustamante VH, Martinez LC, Santana FJ, Knodler LA, Steele-Mortimer O, et al. (2008) HilD-mediated transcriptional cross-talk between SPI-1 and SPI-2. Proc Natl Acad Sci U S A 105: 14591-14596.
5. Kowarz L, Coynault C, Robbe-Saule V, Norel F (1994) The Salmonella typhimurium katF (rpoS) gene: cloning, nucleotide sequence, and regulation of spvR and spvABCD virulence plasmid genes. J Bacteriol 176: 6852-6860.
6. Guzman LM, Belin D, Carson MJ, Beckwith J (1995) Tight regulation, modulation, and high-level

expression by vectors containing the arabinose PBAD promoter. J Bacteriol 177: 4121-4130.
7. Cameron AD, Stoebel DM, Dorman CJ (2011) DNA supercoiling is differentially regulated by environmental factors and FIS in *Escherichia coli* and *Salmonella enterica*. Mol Microbiol 80: 85-101.
8. Hautefort I, Proenca MJ, Hinton JC (2003) Single-copy green fluorescent protein gene fusions allow accurate measurement of *Salmonella* gene expression in vitro and during infection of mammalian cells. Appl Environ Microbiol 69: 7480-7491.
9. Cho BK, Knight EM, Palsson BO (2006) PCR-based tandem epitope tagging system for *Escherichia coli* genome engineering. Biotechniques 40: 67-72.
