# Supplementary material for: A Fundamental Regulatory Mechanism Operating through OmpR and DNA Topology Controls Expression of Salmonella Pathogenicity Islands SPI-1 and SPI-2
Source: PLoS Genet. 2012 Mar 22;8(3):e1002615. doi: 10.1371/journal.pgen.1002615 (PMC3310775; doi:10.1371/journal.pgen.1002615)
Supplement: Table S2 — Oligonucleotide primers used in this study. The table reports the DNA sequences of primers used for cloning, quantitative PCR, mutant construction, DNase I footprinting or electrophoretic mobility shift assays (bandshifts). (PDF) [file pgen.1002615.s004.pdf]

Supporting Table 2: Oligonucleotide primers used in this study

CLONING (Restriction enzyme sites used for cloning are underlined)

Cloning in pZec

PompR.NotI.F	5'- ATA GCG GCC GCT CTT CAC GCC AGA G
PompR.R	5'- ATA TCT AGA CGC ATA TCG TCA TCA ACC
PhilC.F	5'- ACG CCC CGG GAC AAA TGA CCT CCT GGA TGC
PhilC.R	5'- ATT ATC TAG AAT TTG TTC GGC TGT TGA AGG
PhilD.F	5'- ATA CCC CGG GCT TGT TAT CGT CTT CTC TTT TG
PhilD.R	5'- ATT ATC TAG AGA CGC TGA TGA CTA TTA CTT AC
PhilA.F	5'- ATA CCC CGG GCA TTC ATA AAA ATG GCG AAC C
PhilA.R	5'- ATT ATC TAG ACT TTT CTG AGC GTA GCA GGG
PompC.F	5'- GGC ACC CGG GAT AAA TCA GCC GGG TGT GTC
PompC.R	5'- GCC GTC TAG AAT GTA GGT CTG GTC GCC ATC
PompF.F	5'- GGA CCC CGG GCA ACG CCA GCT TCT GAT TTT
PompF.R	5'- ACC ATC TAG AGC ATA AGT CTG GTC GGC ATT
PssaB.F	5'- ATA CCC CGG GCC GTT TCT GAA CCA TTG AT
PssaB.R	5'- ATT ATC TAG ATC CAG GAT CGG CTA TTA CCT T
PsseA.F	5'- ATA CCC CGG GTC TGA GTG CCG ATG TGG TAA
PsseA.R	5'- ATT ATC TAG ACT CAC CTT AGC CCG GAT TT
PssaG.F	5'- ATA CCC CGG GTT TTG GGC GGA CAG ATT TTA
PssaG.R	5'- ATT ATC TAG ACG ATT CTG GGT TGA GCA AAT
PssaM.F	5'- ATA CCC CGG GAA CTT GGC GAA GTG GAT A
PssaM.R	5'- ATT ATC TAG AAG GCC GTT CAG CTA ATC CTG

Cloning in pET21-d

ompR-C.His-NcoI.F	5'- CGA CCC ATG GCT CAA GAG AAT TAT AAG ATT CTG
ompR-C.His-XhoI.R	5'- CGC ACT CGA GTG CTT TAG AAC CGT CCG GTA C

Cloning in pBAD and pUC18 (the *ompB* ribosome binding site is in bold text)

ompR-pBAD-SacI.F	5'- GTT GGA GCT CTT TG**G GAG** TAC AGA CAA TGC AAG AG
envZ-PstI.R	5'- AGC GCT GCA GTG TTC ATT GAG AAC TTC GAG

QUANTITATIVE PCR

Gene expression

ssrA.RT.F	5'- ACA TAA ACG GCA AAG GAT GGC ACG
ssrA.RT.R	5'- TCG GGA AGT TTA ACC GTC ACC TCA
ompR.RT.F	5'- ATC GTC TGC TGA CCC GTG AAT CTT
ompR.RT.R	5'- TTA CTT TGA CTA CGC AGG CGA CGA
hilC.RT/ChIP.F	5'- TGG CAT GTC CAC GGG TTT GTA GTA
hilC.RT/ChIP.R	5'- AGG AAA TCA AAC CCA CCG GGC TTA
hilD.RT.F	5'- TGA GTA CGT CAA CGC TCA AAC GGA
hilD.RT.R	5'- TTG CTG CCT GAT TCA TTC TTG CCG
hilA.RT.F	5'- ATA TGC CGT TCT GGT CAT CCT GCT
hilA.RT.R	5'- AGC CCT GTC CGT ACA GTG TTT CAA

Chromatin immuno-precipitation

ssrA.ChIP.F	5'- ACA GGC AAC TGG AGG GAA GCA TTA
ssrA.ChIP.R	5'- AAG CTG CGG TAA GCA CAG ATA GCA
ompR.ChIP.F	5'- TGC GAA CCT TTG GGA GTA CAG ACA
ompR.ChIP.R	5'- AAG ATT CAC GGG TCA GCA GAC GAT
hilD.ChIP.F	5'- ATT ACC GCA CAG GAC ACA GGG ATT
hilD.ChIP.R	5'- GCG TGT GTT GGC AAT GGT CTG ATT
hilA.ChIP.F	5'- ATA TTT AGA TGC CCG GCG CTG ACT
hilA.ChIP.R	5'- TGT CAT ATT CTA CGG GCT CGG GTT
ompC.ChIP.F	5'- TGA TGG CCT GCA CTA CTT CTC TGA
ompC.ChIP.R	5'- TTT CGC CTT TGA AGC CGA TAC GCA
proV.ChIP.F	5'- CAA TAT TCA TGC CAG AAG CAA A
proV.ChIP.R	5'- CCA TGC AAT AGA ATG ATT CCT G
bamA.ChIP.F	5'- TTC GGT ATC GGT TAC GGT ACA
bamA.ChIP.R	5'- CGT CAA CGG TAA AGT ATG GGT TA

MUTANT CONSTRUCTION

Deletion mutants (the *envZ* start codon is underlined)

fis-KOpKD4.F	5'- GAA AAT TTT GCG TAA ACA GAA ATA AAG AGC TGA CAG AAC TGT GTA GGC TGG AGC TGC TTC
fis-KOpKD4.R	5'- CCG AGT AGC GCC TTT TTA AAC AAG CAG TTA GCT AAT CGA AAC ATA TGA ATA TCC TCC TTA
ompR-KOpKD4.F	5'- ACA CAC TTA CAT TTG TTG CGA ACC TTT GGG AGT ACA GAC AGT GTA GGC TGG AGC TGC TTC
ompR-KOpKD4.R	5'- GGG CAA ATG AAC TTC GCG GTG AGA AGC GCA TTC GCC TCA TCA TAT GAA TAT CCT CCT TAG
envZ-KOpKD4.F	5'- CTA CGT CTT TGT ACC GGA CGG TTC TAA AGC ATG AGG CGA AGT GTA GGC TGG AGC TGC TTC
envZ-KOpKD4.R	5'- GGC GTT GAG AAG AAA GGG AGG GTA ATA CCT CCC TTT CTT ACA TAT GAA TAT CCT CCT TAG
ssrAB-KOpSUB11.F	5'- GTG CCA AAG ATT TTG CAA CAG GCA ACT GGA GGG AAG CAT TGA CTA CAA AGA CCA TGA CGG
ssrAB-KOpSUB11.R	5'- AAT ATG ACC AAT GCT TAA TAC CAT CGG ACG CCC CTG GTT ACA TAT GAA TAT CCT CCT TAG

The *ompB* locus was deleted by combining primers ompR-KOpKD4.F and envZ-KOpKD4.R

Epitope tagging

ompR-Flag.F	5'- GGG CCT GGG CTA CGT CTT TGT ACC GGA CGG TTC TAA AGC AGA CTA CAA AGA CCA TGA CGG
ompR-Flag.R	5'- GGG CAA ATG AAC TTC GCG GTG AGA AGC GCA TTC GCC TCA TCA TAT GAA TAT CCT CCT TAG
fis-Myc.F	5'- GGT ACG CTG CGT AAA AAA TTA AAA AAA TAC GGC ATG AAC GTC GGA TCC AGT CTT CGT GAT
fis-Myc.R	5'- GAG TAG CGC CTT TTT AAA CAA GCA GTT AGC TAA TCG AAA AAT TCC GGG GAT CCG TCG ACC

Point mutation of ompR

ompR-D55E.F	5'- CCA TCT CAT GGT ACT GGA ATT AAT GCT GCC AGG TGA AGA TGG T
ompR-D55E.R	5'- ACC ATC TTC ACC TGG CAG CAT TAA TTC CAG TAC CAT GAG ATG G

DNASE I FOOTPRINTING AND BANDSHIFT

pZec-6FAM.F	5'- 6-FAM/AC AAA TCC GCC CTC TAG CAG CCC G
pZec-6FAM.R	5'- 6-FAM/CT CCT TTA CTC ATA TGT ATA TCT CC
pZec-confirm.F	5'- CTG AGT AGG ACA AAT CCG C
pZec-confirm.R	5'- ACG GGA AAA GCA TTG AAC AC
